# A Genomic Catalog of Stress Response Genes in Anaerobic Fungi for Applications in Bioproduction

**DOI:** 10.3389/ffunb.2021.708358

**Published:** 2021-08-09

**Authors:** Candice L. Swift, Nikola G. Malinov, Stephen J. Mondo, Asaf Salamov, Igor V. Grigoriev, Michelle A. O'Malley

**Affiliations:** ^1^Department of Chemical Engineering, University of California, Santa Barbara, Santa Barbara, CA, United States; ^2^U.S. Department of Energy Joint Genome Institute, Lawrence Berkeley National Laboratory, Berkeley, CA, United States; ^3^Department of Agricultural Biology, Colorado State University, Fort Collins, CO, United States; ^4^Lawrence Berkeley National Laboratory, Environmental Genomics and Systems Biology Division, Berkeley, CA, United States; ^5^Department of Plant and Microbial Biology, University of California, Berkeley, Berkeley, CA, United States; ^6^Joint BioEnergy Institute, Emeryville, CA, United States

**Keywords:** UPR, HSR, transcriptomics, anaerobic fungi, stress response, chaperones, protein folding

## Abstract

Anaerobic fungi are a potential biotechnology platform to produce biomass-degrading enzymes. Unlike model fungi such as yeasts, stress responses that are relevant during bioprocessing have not yet been established for anaerobic fungi. In this work, we characterize both the heat shock and unfolded protein responses of four strains of anaerobic fungi (*Anaeromyces robustus, Caecomyces churrovis, Neocallimastix californiae*, and *Piromyces finnis*). The inositol-requiring 1 (Ire1) stress sensor, which typically initiates the fungal UPR, was conserved in all four genomes. However, these genomes also encode putative transmembrane kinases with catalytic domains that are similar to the metazoan stress-sensing enzyme PKR-like endoplasmic reticulum kinase (PERK), although whether they function in the UPR of anaerobic fungi remains unclear. Furthermore, we characterized the global transcriptional responses of *Anaeromyces robustus* and *Neocallimastix californiae* to a transient heat shock. Both fungi exhibited the hallmarks of ER stress, including upregulation of genes with functions in protein folding, ER-associated degradation, and intracellular protein trafficking. Relative to other fungi, the genomes of Neocallimastigomycetes contained the greatest gene percentage of HSP20 and HSP70 chaperones, which may serve to stabilize their asparagine-rich genomes. Taken together, these results delineate the unique stress response of anaerobic fungi, which is an important step toward their development as a biotechnology platform to produce enzymes and valuable biomolecules.

## Introduction

The ability to cope with different kinds of environmental stress is a universal trait of life. However, depending on the type of stress, the response can be highly conserved between organisms, such as the heat shock response (HSR) (Martin and Gretchen, 1999), or the response can vary significantly between the kingdoms of life, such as in the case of the unfolded protein response (UPR) (Hollien, 2013). Heat shock causes both cytosolic and ER stress, triggering the HSR and potentially the UPR (Mager and Ferreira, 1993), in which cells respond to misfolded proteins by attenuating protein production. In biotechnology, cellular stress responses can decrease product titer or even cause cell death. Fungi have adapted to cope with many kinds of stressors in their native environments. Heat shock proteins (hsps) are used to respond to cytosolic stress triggered by various stimuli, including heat (Boreham and Mitchel, 1994; Glover and Lindquist, 1998; Borchsenius et al., 2001), osmotic pressure (Fernandes et al., 2004), and low pH (Estruch and De Valencia, 2001; Burnie and Matthews, 2006). The expression of hsps in the HSR as well as the UPR are part of the comprehensive response of an organism, called the environmental stress response, to various types of environmental stresses (Gasch and Werner-Washburne, 2002; Gasch, 2007). The environmental stress response is well-established for Ascomycota, especially for *Saccharomyces cerevisiae* (Gasch et al., 2000; Causton et al., 2001). Both the HSR and UPR are often triggered during expression of proteins, especially those that are a product of heterologous expression efforts (Mattanovich et al., 2004; Guillemette et al., 2007; O'Malley et al., 2009).

Although the UPR is induced by stress to the ER rather than the cytosol, some features of the UPR and HSR are shared, such as the upregulation of genes with functions in polypeptide translocation, vesicular transport from the ER, and ER-Associated Degradation (ERAD) to increase protein-folding capacity and to limit protein production (Liu and Chang, 2008). Although the UPR is found in all eukaryotes, the cellular machinery involved in the response differs between the kingdoms of life (Hollien, 2013). The unfolded protein response of fungi is well-established, especially for yeasts and filamentous fungi applied in biotechnology (Travers et al., 2000; Guillemette et al., 2007; Jung et al., 2016; Hernández-Elvira et al., 2018) as well as pathogenic fungi (Heimel et al., 2013; Cheon et al., 2014; Starke et al., 2021). Fungi are known to possess the transmembrane endoribonuclease Ire1, which senses misfolded proteins, oligomerizes and autophosphorylates, activating an endoribonuclease domains that removes a constitutively expressed intron from mRNA encoding the transcription factor Hac1 (Cox and Walter, 1996; Mori et al., 1996). This transcription factor triggers the upregulation of genes encoding chaperones to promote protein folding and those with functions in vesicular transport and protein turnover (Cox et al., 1993; Morl et al., 1993; Travers et al., 2000). In contrast, members of Metazoa use both the Ire1-initiated pathway as well as two others: ATF6 and PERK (Gardner et al., 2013; Hollien, 2013). PKR-like Endoplasmic Reticulum Kinase (PERK) is also a transmembrane sensing protein, but instead of cleaving an intron, it phosphorylates the eukaryotic initiation factor 2 alpha subunit (eIF2α), which prohibits GTP exchange and subsequent delivery of Met-tRNA to initiate translation (Dever, 2002; Wek et al., 2006; Wek and Cavener, 2007). This event results in global inhibition of protein translation, except for certain proteins (Gardner et al., 2013).

PERK is not the only kinase of eIF2α that regulates translation in eukaryotes. Different kinases of eIF2α evolved to sense diverse types of environmental stress: ER stress (PERK); nutrient limitation (GCN2); viral infection (PKR/PKZ); and heme deprivation, heat shock, or oxidative stress (HRI) (Hernández and Jagus, 2016). The domain architecture for each kinase is distinct (Hernández and Jagus, 2016). The only eIF2α kinase with a transmembrane domain is PERK. The archetypical PERK also contains an N-terminal signal peptide, IRE1-like stress sensing and dimerization domain, and a catalytic kinase domain with an internal insert (Hernández and Jagus, 2016). GCN2 is ubiquitously present in fungi, whereas HRI is not found in all fungi. To date, no instance of a PERK-like kinase has been reported in fungi (Hernández and Jagus, 2016).

Understanding how to circumvent cellular stress responses facilitates fungal bioprocessing, especially heterologous protein production, as illustrated by previous studies of filamentous fungi (Heimel, 2014) and yeasts (Valkonen et al., 2003a; Mattanovich et al., 2004; Gasser et al., 2007). With appropriate genetic engineering strategies, knowledge of UPR components can be leveraged for enhanced production of secreted proteins to improve product titers, without the induction of deleterious effects such as ER-Associated Degradation (ERAD) (Heimel, 2014). One successful example was the sevenfold boost in production of laccase by *Aspergillus niger* var. *awamon* as a result of the overexpression of active *hacA* transcription factor (Valkonen et al., 2003b). Similarly, constitutive overexpression of *HAC1* in *Saccharomyces cerevisiae* resulted in a 70% increase in the secretion of foreign protein α-amylase from *Bacillus amyloliquefaciens* (Valkonen et al., 2003a). However, this approach is not successful for all heterologous production schemes. For example, constitutive overexpression of *HAC1* in *Saccharomyces cerevisiae* did not increase the secretion of the foreign protein endoglucanase from *Trichoderma reesei* (Valkonen et al., 2003a). Other factors, such as glycosylation and proper folding temperature may impact the success of heterologous production schemes. For example, *N-*linked glycosylation stabilizes an endoglucanase from *Penicillium* verruculosum against proteolytic attack when expressed recombinantly in *Penicillium* canescens (Dotsenko et al., 2016).

Our knowledge of the fungal stress response has largely been limited to Dikarya (higher-order fungi). Neocallimastigomycetes, a clade of the phylum Chytridiomycota, are early-diverging fungi native to the digestive tracts of large herbivores. They specialize in the degradation of plant biomass and possess the largest array of carbohydrate active enzymes (CAZymes) of any sequenced fungi to date and thus hold great promise for applications in biotechnology (Solomon et al., 2016a; Seppälä et al., 2017; Henske et al., 2018). Recent improvements in anaerobic fungal cultivation (Haitjema et al., 2014; Solomon et al., 2016b) and the sequencing of high-quality genomes and transcriptomes (Haitjema et al., 2017) have advanced our understanding of these non-model organisms, but the HSR and UPR have not been characterized.

Here, we delineate several major components of the UPR, including the key stress-sensing enzymes, chaperones, and some target genes associated with the secretory pathway. We further validate the identified components of the HSR and UPR within Neocallimastigomycetes by subjecting two representative strains from this class (*Anaeromyces robustus* and *Neocallimastix californiae*) to heat shock and subsequently tracking their transcriptomic response. Moreover, we report on and quantify an unusual prevalence of small heat shock proteins within the genomes of Neocallimastigomycetes relative to other fungi, which may have implications in the stability of their proteomes.

## Results and Discussion

### Neocallimastigomycetes May Share Components of the Metazoan Unfolded Protein Response

We delineated components of the UPR and target genes of the secretory pathway in four strains of Neocallimastigomycetes (*Anaeromyces robustus, Caecomyces churrovis, Neocallimastix californiae*, and *Piromyces finnis*) by homology to model organisms (Supplementary Table 1). Homologs of Ire1 were identified in all four genomes, as well as the critical chaperones KAR2, calnexin, and calreticulin, and the catalyst of disulfide bond formation ERO1 (ER oxidoreductin 1). In most cases, the genes were highly conserved between Neocallimastigomycetes and *S. cerevisiae*, with percent identities and coverages >40 and 80%, respectively (Supplementary Table 1). *S. cerevisiae* only possesses a single gene with similarity to the calnexin of mammalian cells, rather than separate calnexin and calreticulin proteins (Parlati et al., 1995). In contrast, homologs of *S. cerevisiae* calnexin and *D. melanogaster* calreticulin were identified in all four Neocallimastigomycetes (Supplementary Table 1). Additional components of the secretory pathway were previously identified in other anaerobic fungi: components of the gamma-secretase complex in *Orpinomyces* sp. (now *Pecoramyces*) (Youssef et al., 2013), and endoplasmic reticular translocon family proteins, SNAREs (Synaptosomal Vesicle Fusion Pore proteins). Synaptic Vesicle Associated Calcium Channels. and Annexin-like Proteins in *A. robustus, N. californiae*, or *Piromyces finnis* (Seppälä et al., 2016).

**Figure 1:**
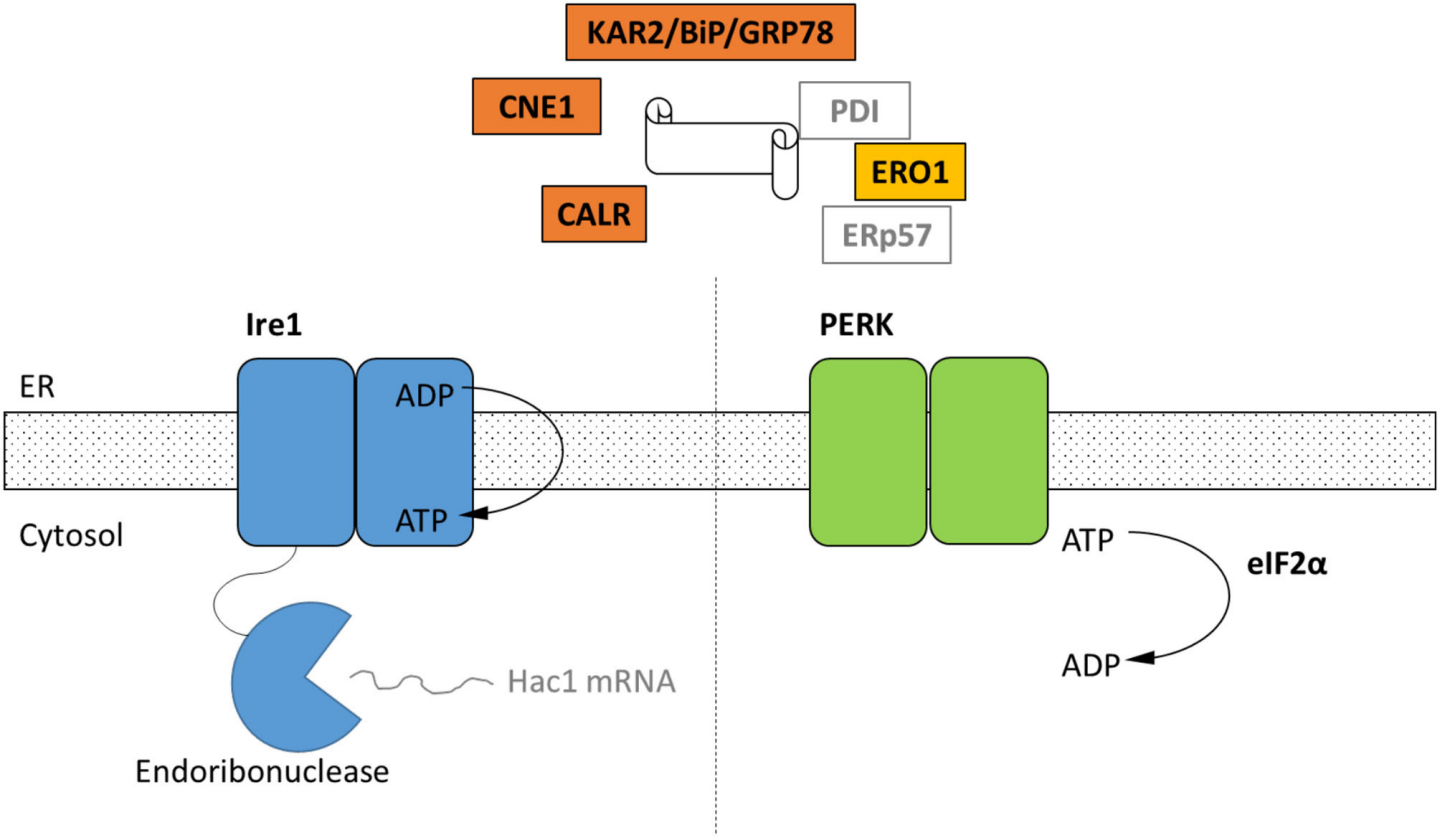
Schematic of the cellular components of the UPR identified by homology only within the genomes of four representative strains of Neocallimastigomycetes. Black text indicates homologs of UPR components that were identified in in the genomes of *A. robustus*, C. *churrovis, N. californiae*, and *P. finnis* (Supplementary Table 1) whereas gray text signifies parts not identified. Ire1 and PERK are two transmembrane receptors of misfolded proteins. Ire1 activates the Hac1 transcription factor through an alternative splicing event. In the metazoan UPR, PERK oligomerizes and then phosphorylates eIF2α, resulting in global translational repression, which has not been demonstrated for anaerobic fungi. KAR2/BiP/GRP78 functions in protein translocation and folding. Following translocation into the lumen, chaperones KAR2 and lectins calnexin (CNE1) and calreticulin (CALR) work to fold the polypeptide. Disulfide bond formation is achieved through Ero1, protein disulfide isomerase (PDI), and ERp57.

**Figure 2:**
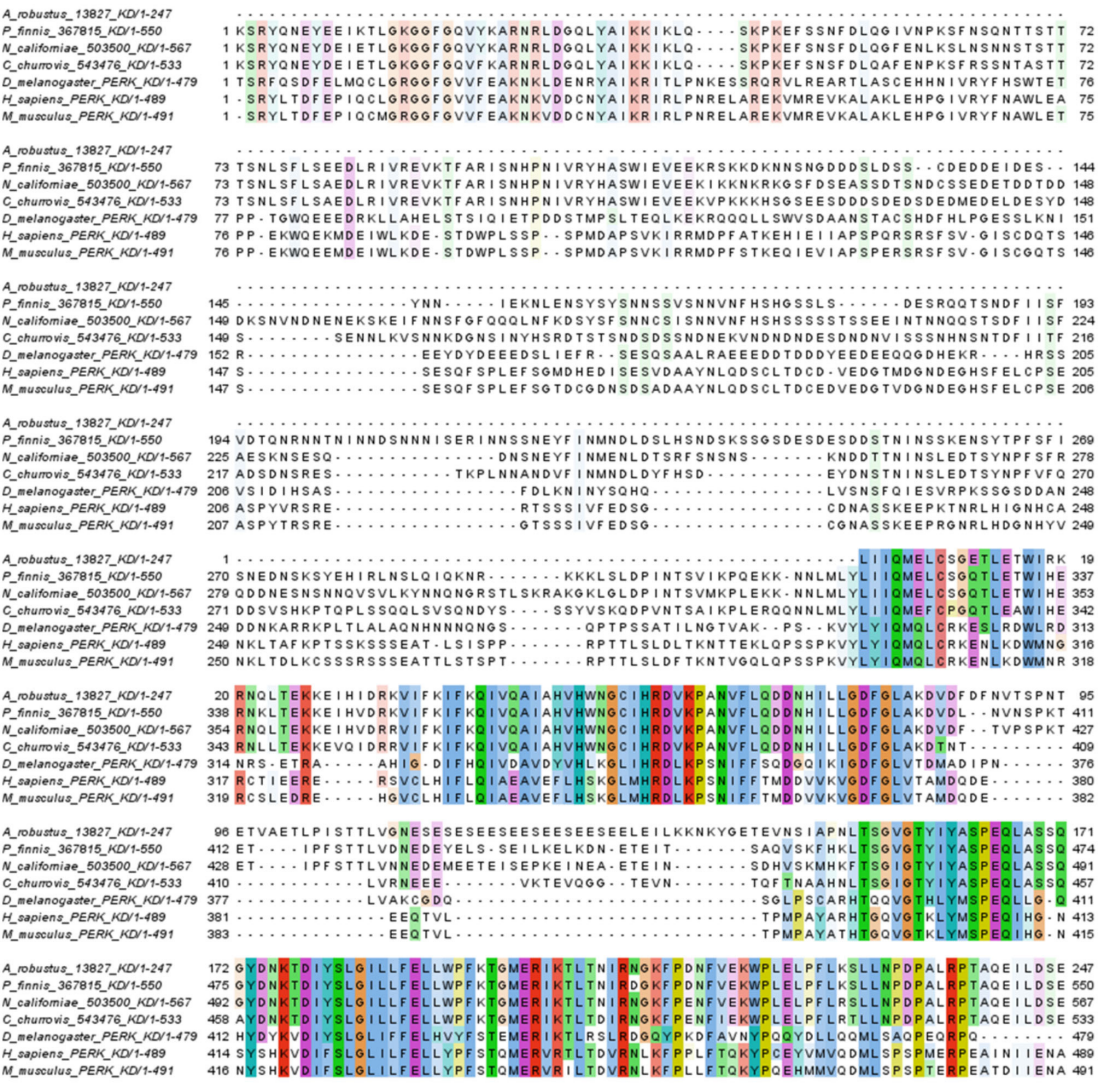
Conserved residues between kinase domains of PERK-like proteins from Neocallimastigomycetes and representative kinase domains from metazoan PERK proteins. Black bars indicate protein kinase catalytic domains [Conserved Domain Database (Marchler-Bauer et al., 2014) accession cl21453]. Coloring follows Clustal X designations for amino acid properties: Blue, hydrophobic; red, positive; magenta, negative; green, polar; pink, cysteine; orange, glycine; yellow, proline; cyan, aromatic. A threshold of 20% conservation was used to set transparency.

Surprisingly, protein sequences similar to the metazoan PERK transmembrane protein were also identified (Figure 1; Supplementary Table 1). Closer inspection of the PERK-like sequences in Neocallimastigomycetes showed sequence similarity only in the catalytic domain (Figure 2) and not the luminal dimerization domain. Although the presence of an additional UPR pathway in fungi is unprecedented, kinases of eIF2α that respond to ER stress have been identified in *Toxoplasma gondii* (Narasimhan et al., 2008), which is also an early eukaryote.

**Figure 3:**
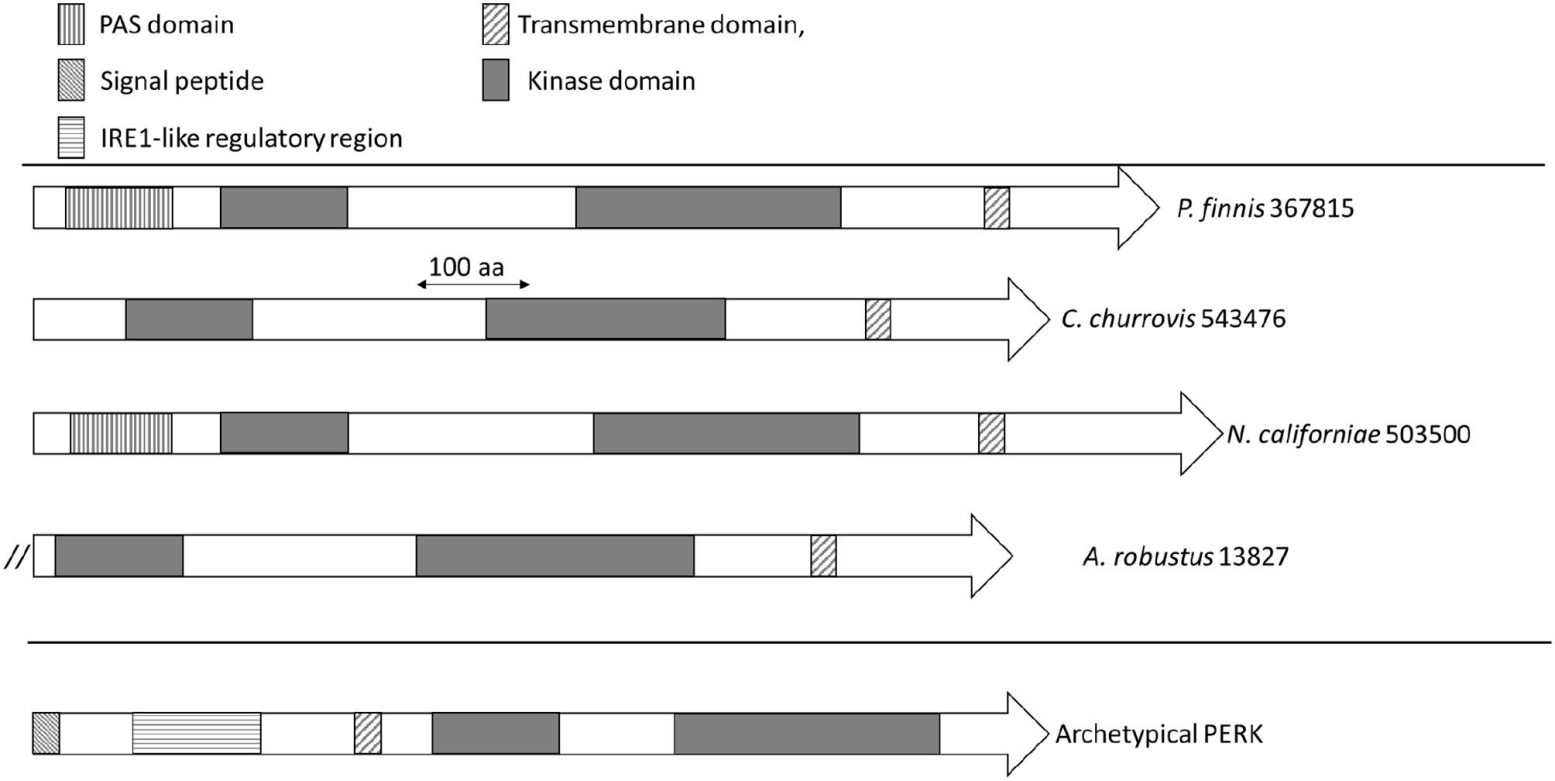
Domain architecture of PERK and PERK-like kinases from Neocallimastigomycetes. Domains are indicated by rectangles, with the significance of the patterns depicted in the legend. The double forward slash for *A. robustus* 13827 indicates the possible truncation of this gene model due to its location on a scaffold end.

The domain architecture of PERK candidates from Neocallimastigomycetes (Supplementary Table 1) is illustrated in Figure 3. *N. californiae* 503500 and *P. finnis* 367815 both contained N-*terminal* PAS domains. *A. robustus* 13827 is located at the start of a scaffold, which indicates that the gene model may be truncated. Thus, the full gene may contain a PAS domain. PAS domains are known sensing modules of signal transduction proteins, such as kinases (Taylor and Zhulin, 1999; Amezcua et al., 2002). *A. robustus* 13827 only contained the catalytic domain and no PAS domain. SignalP-5.0 (Almagro Armenteros et al., 2019) predicted no signal peptides in the PERK-like kinases from Supplementary Table 1. However, TMHMM2.0 (Krogh et al., 2001) identified a transmembrane helix located near the C-terminus in all sequences, which is inconsistent with PERK found in Metazoa. Sequence alignment of PERK-like kinases from Neocallimastigomycetes to representative members of Apicomplexa, including the TgIF2K-A kinase from *T. gondii* that responds to ER stress, only demonstrated consensus in the kinase domain regions. However, sequence alignment by Clustal Omega (Sievers et al., 2011) and visualization with Jalview (Waterhouse et al., 2009) of *P. finnis* 367815, *N. californiae* 503500, and *C. churrovis* 543476 indicated a high degree of similarity, even in regions outside of the kinase domain (Supplementary Figure 1).

At present, the function of these transmembrane kinases of anaerobic fungi remains unclear given the similarity in the catalytic domain to metazoan PERK but the atypical domain architecture. One possibility is that these kinases may sense another form of stress in the cytoplasm, such as oxidative stress, rather than misfolded proteins in the ER. HRI is typically the eIF2alpha kinase that responds to oxidative stress (Rothenburg et al., 2016) and has been found in fungi such as *Shizosaccharomyces pombe* (Martin and Gretchen, 1999). The transmembrane kinases from anaerobic fungi in Figure 3 lack the heme-binding domains that are required for function in HRI, but it is possible that the PAS domain serves to sense oxidative stress or redox changes, which is the case in some bacteria (Taylor and Zhulin, 1999).

### Comparative Transcriptomics of the Responses of *A. robustus* and *N. californiae* to Heat Shock Affirms Signature Stress Response Genes

Neocallimastigomycetes are highly sensitive to temperature fluctuations, most likely due to the fact that they are native to the rumen, which is tightly temperature-controlled at 39°C (Trinci et al., 1994). In the laboratory, rumen fungi grow optimally at 39–42°C (Orpin, 1975, 1976). To capture the transcriptomic response to heat shock, without inducing cell death, we measured fungal growth curves at a range of heat shock temperatures and for varying durations to test an optimal temperature shift and duration that would limit, but not completely suppress, growth (Supplementary Figure 2). We found that temperature shock at 48°C for 15 min met these criteria.

**Figure 4:**
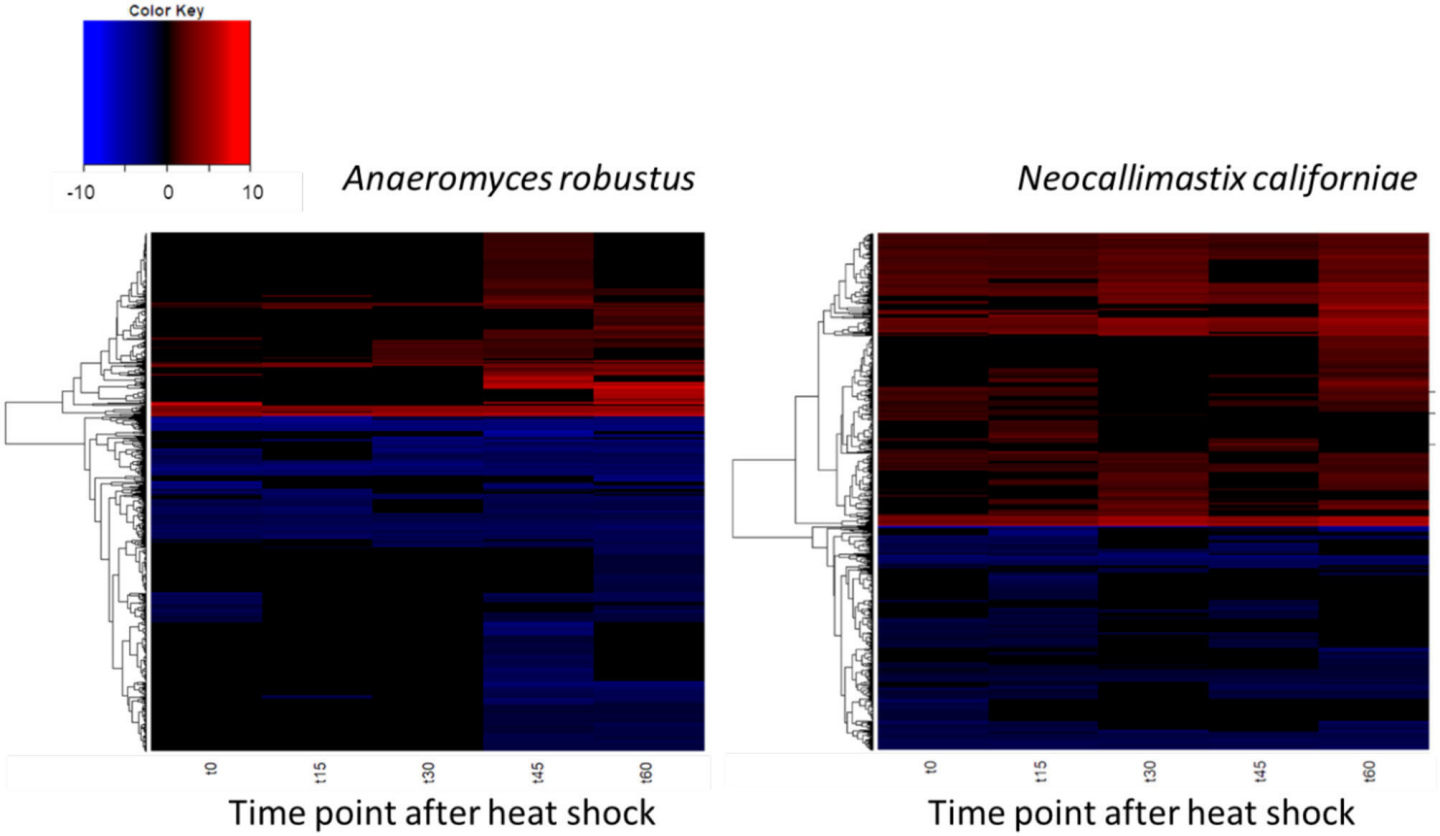
Dynamics of the transcriptomic responses to heat shock differ between *A. robustus* and *N. californiae*. Each row represents the log_2_ fold change of a transcript relative to the control without heat shock. Only transcripts with absolute log_2_FC greater than or equal to one and adjusted *p*-value ≤ 0.05 are shown. X-axis labels for each heat map are as follows: “t0” represents time zero, immediately after the 15 min heat shock, “t15” represents 15 min after t0 time point, and so forth up to 60 min after the completion of the heat shock (“t60”). Plots were rendered using the “heatmap.2” function in the “ggplots” package of R software version 3.4.3 (R Core Team, 2013).

The global transcriptomic responses of *A. robustus* and *N. californiae* to a 15-min duration heat shock at 48°C were captured, with the dynamics of the responses measured by time points at 15-min intervals up to 1 h after stress was induced. Differentially regulated genes were identified as those with an absolute log_2_ fold change greater than one compared to a control condition without heat shock harvested immediately prior to the heat shock of the test cultures (*p*-adjusted <0.05). The count of up- and downregulated genes at each time point indicates that the greatest response, as measured by the number of differentially regulated genes and the magnitude of the largest log_2_fold change, occurred 45–60 min after heat shock (Figure 4; Supplementary Figure 3). A complete list of differentially regulated genes at each time point can be found in Supplementary Datasets 1, 2.

**Figure 5:**
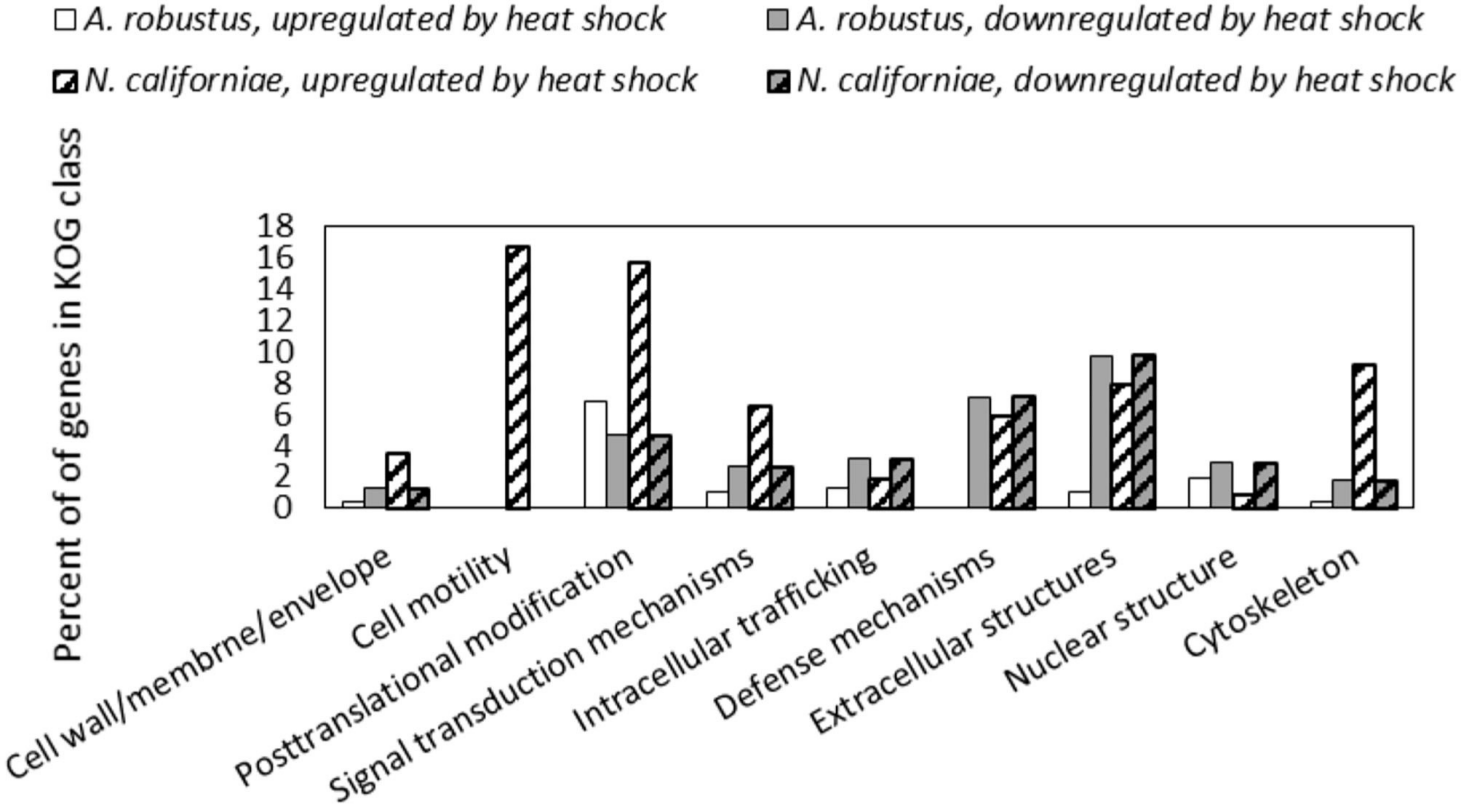
Differential regulation in response to heat shock of *A. robustus* and *N. californiae* genes in each class belonging to the eukaryotic Orthologous Group (Koonin et al., 2004) (KOG) Cellular Processes and Signaling. Top: *A. robustus*, bottom: *N. californiae*. Genes were counted as differentially regulated only for those with an absolute log_2_FC ≥1 (*p*-adjusted ≤ 0.05) 1 h after completion of heat shock compared to the control without heat shock. The number of gene models assigned to each KOG class, excluding CAZymes are as follows (*A*. robustus; *N*. californiae): Cell wall/membrane/envelope (235; 313), Cell motility (11; 18), Posttranslational modification (770; 1,240), Signal transduction mechanisms (1,008; 1,452), Intracellular trafficking (479; 871), Defense mechanisms (112; 169), Extracellular structures (92; 101), Nuclear structure (104; 225), Cytoskeleton (281; 501). KOG assignments in the MycoCosm portal were used for all gene annotations.

In both *A. robustus* and *N. californiae* analysis of the eukaryotic Orthologous Groups (KOGs) (Koonin et al., 2004) revealed that the proportion of differentially regulated genes was highest in the group Cellular Processes and Signaling (Figure 5). Posttranslational modification, protein turnover, chaperones was the class with the most differentially expressed genes 1 h after heat shock compared to before heat shock. Most of the upregulated genes within this KOG class were chaperones belonging to the HSP20 or HSP70 families (Supplementary Datasets 1, 2). For *A. robustus* 7% of the total genes classified in this KOG class were at least 2-fold upregulated and for *N. californiae* 16% were upregulated (*p*-adjusted <0.05). This finding supports that HSR was activated in *A. robustus* and *N. californiae* upon exposure to thermal stress at 48°C. Further supporting that a stress response was activated, *A. robustus* upregulated 12 of its 15 genes encoding glutamate dehydrogenase (E.C. 1.4.1.4) at least 2-fold (*p*-adjusted <0.05) 1 h after heat shock. In *Saccharomyces cerevisiae*, glutamate dehydrogenase has been linked to the phenotype of resistance to thermal and oxidative stress-induced apoptosis (Lee et al., 2012). The upregulation of multiple copies of glutamate dehydrogenase implies that glutamate dehydrogenase may perform a similar function in *A. robustus*.

To verify whether ER stress was also induced, we searched for differentially regulated genes that indicated changes in protein folding and an increased burden of misfolded proteins. Previous work in model ascomycetes (Guillemette et al., 2007) indicated that genes with functions in protein traffic and secretion are upregulated in response to denaturants such as dithiothreitol (DTT) and tunicamycin, which are frequently used to elicit UPR (Guillemette et al., 2007; Liu and Chang, 2008). However, HSR also targets genes related to the secretory pathway and can relieve ER stress (Liu and Chang, 2008). We found that both *N. californiae* and *A. robustus* significantly upregulated genes within the KOG class Intracellular trafficking, secretion, and vesicular transport (Supplementary Tables 2, 3). Retrograde transport from the Golgi to the ER in particular was upregulated, as evidenced by the upregulation of genes encoding putative COPI subunit proteins (Supplementary Table 3). Consistent with the accumulation of misfolded proteins associated with ER stress, both *N. californiae* and *A. robustus* upregulated genes involved in the protein degradation, such as ubiquitination enzymes and proteasomes (Supplementary Tables 2, 3). Other key players of the secretory pathway that are implicated in the UPR include ER oxidoreductin (ERO1) and the hsp70 chaperone KAR2 (Kaufman et al., 2002). One of the *N. californiae* homologs of KAR2, MycoCosm (Grigoriev et al., 2014) protein Id 377732, was upregulated 60 min after heat shock by a log_2_ fold change of 1.47 relative to the control without heat shock (*p*-adjusted <0.05).

*A. robustus* differentially regulated a total of 66 genes assigned to the KOG class Signal transduction mechanisms at one or more time points relative to the control without heat shock. Nine of the upregulated genes were annotated as Serine/threonine protein kinases (KOG1187). Since the protein kinase Hog1 in *S. cerevisiae* initiates the environmental stress response (Brewster et al., 1993; Gasch, 2007), we wondered whether any of these protein kinases were homologs. In *A. robustus*, protein Id 197439 was upregulated 2-fold (*p*-adjusted ≤ 0.05) at 30, 45, and 60 min after heat shock vs. the no heat shock control. Protein BLAST (Altschul et al., 1990) alignment of *A. robustus* protein Id 197439 from MycoCosm (Grigoriev et al., 2014) to *S. cerevisiae* Hog1 (accession NP_013214) resulted in 48% identity and 95% coverage between the sequences (E-value 4e-108). These findings suggest that this gene may be evolutionarily related to the Hog1 of higher order fungi. However, there was no corresponding homolog in *N. californiae* that was upregulated in response to heat shock, although protein Ids 424919, 409946, and 523809 were identified as top hits when *A. robustus* 197439 was searched against all filtered model proteins of *N. californiae* using BLAST+ (Camacho et al., 2009).

Within the group Information Storage and Processing, *A. robustus* upregulated 6% of genes in the class Chromatin structure and dynamics. N6-adenine methylation has been shown to mark transcriptionally active genes in early-diverging fungi (Mondo et al., 2017). In *A. robustus* the gene encoding protein Id 282395, containing an N6-adenine-specific DNA methylase domain (IPR002052) was upregulated by more than 32-fold 60 min after heat shock compared to the control without heat shock (*p*-adjusted <0.05). This finding suggests that *A. robustus* may epigenetically regulate gene expression in response to heat shock.

### Neocallimastigomycetes Harbor a Disproportionate Number of Small Chaperones Among Fungi

**Figure 6:**
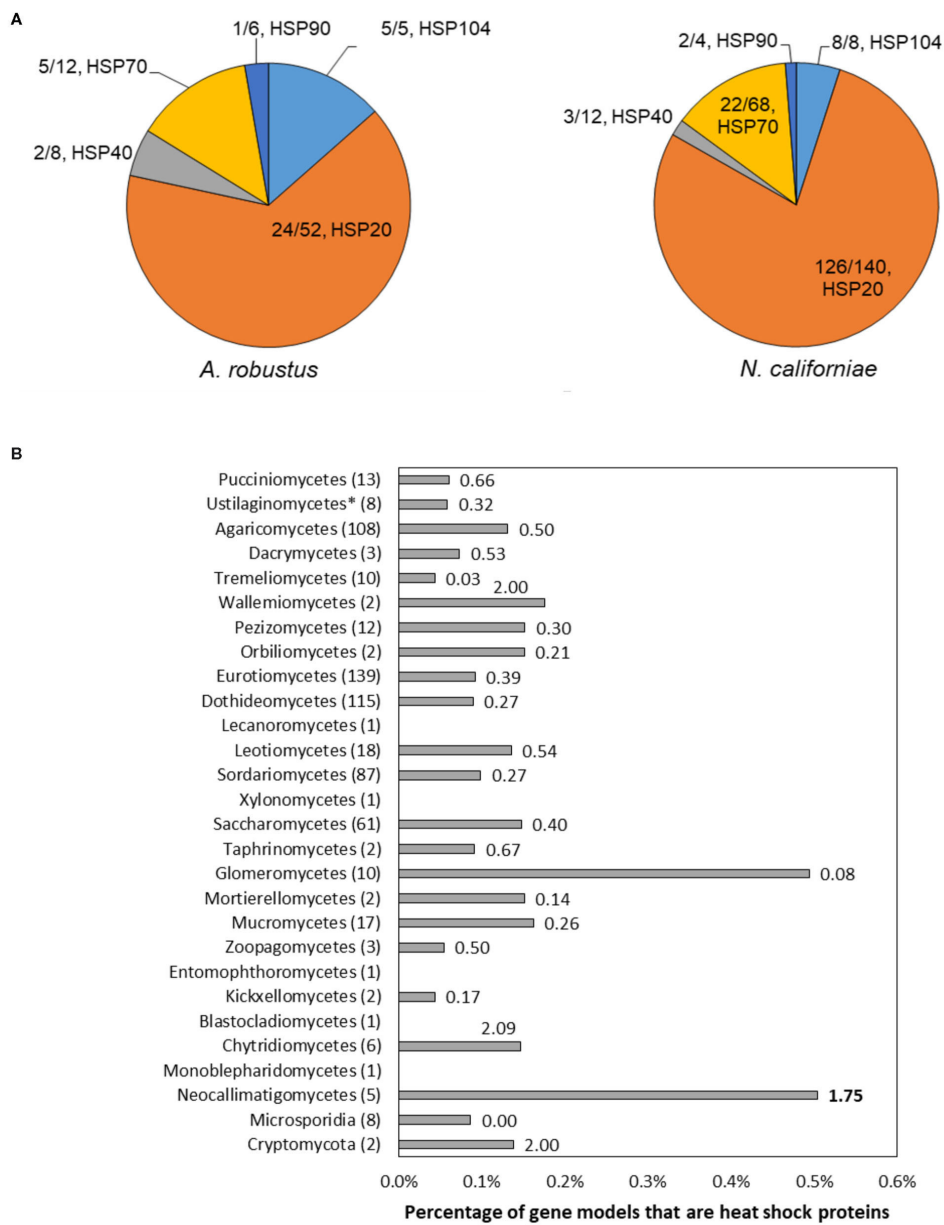
The genomes of Neocallimastigomycetes are enriched in heat shock proteins (hsps) and favor small hsps over large hsps compared to other fungi. **(A)** Pie charts depict the proportion of upregulated genes of each type out of the total number of upregulated hsps (absolute log_2_FC ≥ 1, *p*-adjusted ≤ 0.05) at 45 min (*A. robustus*) or 60 min (*N. californiae*) after the completion of the heat shock. The presented fractions are the number of upregulated hsps in each class divided by the total number of MycoCosm gene models with the corresponding KOG annotation. KOG0710 (HSP20/HSP42) was further annotated by InterPro as HSP20. **(B)** Percentages given are the number of genes annotated as KOG0710 (HSP26/HSP42) or KOG0101 (HSP70/HSC70, HSP70 superfamily) out of the total number of gene models for each genome, averaged over all published genomes from that clade available from the MycoCosm portal. Each data label is the ratio of small hsps to large hsps, calculated from the number of gene models assigned to KOG0710 or KOG0101 within each genome. Ratios were also averaged across all published genomes within a clade. All clades had a significantly different hsp percentage relative to the Neocallimastigomycetes, except for Glomeromycetes, as assessed by a two-tailed, two-sample unequal variance student's *t*-test (alpha level <0.1). Numbers in parenthesis after each clade name indicate the number of genomes represented from that clade. Clades are listed in order of divergence from the earliest common ancestor.

The majority of the upregulated genes encoding heat shock proteins in the response of *A. robustus* and *N. californiae* to heat shock were predicted to be small (~20 kDa, Supplementary Datasets 1, 2). Although many small hsps are sequence divergent, especially between different organisms (Lindquist and Craig, 1988), the upregulation of these putative small hsps in response to heat shock corroborates the sequence-based prediction. Upon inspection of the genomes of Neocallimastigomycetes, we found that the number of small hsps per genome is on the same scale as some plants (>20 small hsps) (Wu et al., 2016). Since the average genome sizes of Neocallimastigomycetes are at least an order of magnitude smaller than plant genomes (Schmuths et al., 2004; Wu et al., 2016; Haitjema et al., 2017), this indicates that Neocallimastigomycetes genomes are relatively enriched in small heat shock proteins. Similarly, hsps are also enriched on a gene count basis, since the number of gene models for *A. robustus, C. churrovis*, and *P. finnis* is less than the model plant *Arabidopsis thaliana* (Itoh et al., 2007). This strong preference for small chaperones is not observed in other fungi, as evidenced in Figure 6. By sampling all published genera available from the MycoCosm portal (Grigoriev et al., 2014) for each clade on the fungal evolutionary tree, we observed that Neocallimastigomycetes have the highest percentage (0.58%) of hsps (size 70 or 20 kDa) out of all predicted genes, as well as six times the number of predicted hsps belonging to the 20 kDa class compared to the number of genes within the 70 kDa class. *A. robustus* and *N. californiae* upregulated more HSP20 chaperones relative to any other hsp class (Figure 6), in line with the overrepresentation of small chaperones in their genomes. The proportion of hsps in each class out of all upregulated hsps was similar between *A. robustus* and *N. californiae*, although the percent utilization of each class differed. For example, *A. robustus* upregulated 46% of the total HSP20 genes and *N. californiae* upregulated 90% of all HSP20 genes. The remainder of the HSP20 genes are likely upregulated by other environmental stressors, which may include pH stress, osmotic stress, or others.

The genomes of rumen fungi are known to be AT-rich and subsequently plentiful in asparagine repeats (Wilken et al., 2020). Similarly, the malaria-causing parasite *Plasmodium falciparum* from the Apicomplexa phylum also has a genome rich in asparagine repeats, and the parasite compensates for the propensity of its proteins to agglomerate by using the heat shock protein 110 and likely other chaperones to stabilization its proteome (Muralidharan et al., 2012; Muralidharan and Goldberg, 2013). It is possible that rumen fungi may use their hsps to stabilize their asparagine-rich proteomes, similar to *Plasmodium falciparum*. Furthermore, the majority of the small hsps of the Neocallimastigomycetes were constitutively transcribed at >0.5 RPKM during normal laboratory cultivation (Supplementary Table 5).

## Conclusion

Knowledge of the stress responses of fungi can inform engineering efforts that improve their application as bioproduction platforms. In particular, the HSR and UPR can be triggered by heterologous protein expression or processing conditions, respectively. In model fungi such as yeasts, engineering the sensor of the UPR has led to improvements in product titer in some cases. Although these responses have been studied in detail in model fungi, little is known about the stress responses of anaerobic fungi. In this work, we cataloged the stress response genes of anaerobic fungi through genomics and transcriptomics. We identified through homology top candidates for genes in the unfolded protein response (UPR). We also measured transcriptional abundance of these genes after heat shock. We demonstrated that in the genomes from three genera of anaerobic fungi, a putative PERK-like protein is encoded. These predicted transmembrane proteins are highly conserved between genera and are homologous in the catalytic kinase domain to metazoan PERK proteins, although their domain architecture is not consistent with PERK. The function of these kinases in anaerobic fungi is unknown and requires experimental validation. We also demonstrated that the genomes of Neocallimastigomycetes are enriched in heat shock proteins (hsps), especially small hsps, compared to all other fungi. The majority of the small hsps are constitutively transcribed during standard laboratory cultivation. These constitutively transcribed hsps may serve to stabilize an asparagine-rich genome, which has been demonstrated to be the case in *Plasmodium falciparum*. By establishing a baseline for the genetics of the UPR and HSR in anaerobic fungi, we have paved the way for future efforts to engineer these pathways to provide enhanced resilience to the anaerobic fungi during production of biomolecules such as carbohydrate-active enzymes.

## Materials and Methods

### Annotation of Genes in the Neocallimastigomycete Unfolded Protein Response

Sequences of components of the UPR in model organisms *Saccharomyces cerevisiae* and *Drosophila melanogaster* were queried against the filtered model proteins of *Anaeromyces robustus, Caecomyces churrovis, Neocallimastix californiae*, and *Piromyces finnis* using protein BLAST (Altschul et al., 1990) within the MycoCosm portal (Grigoriev et al., 2014). Query protein sequences were as follows (accession numbers are given in parenthesis): KAR2 (NP_012500), IRE1 (NP_011946.1), PERK (NP_649538), eIF2alpha (NP_001285329), and, ERO1 (NP_013576), CNE1 (NP_009343), and CALR (NP_001262430). Candidate PERK proteins were also identified by searching the MycoCosm portal (Grigoriev et al., 2014) for filterered model proteins annotated as KOG1033 eIF-2alpha kinase PEK/EIF2AK3. Candidate PERK gene models were checked for RNA coverage in the MycoCosm genomebrowser, using previously published RNA-seq data (Solomon et al., 2016a). *A. robustus* 182287 was selected instead of protein Id 13827 because the associated gene model had better RNA-seq coverage. Each protein sequence was queried for transmembrane helices by TMHMM2.0 (Krogh et al., 2001) and for signal peptides by SignalP-5.0 (Almagro Armenteros et al., 2019). Only proteins with transmembrane helices were considered as PERK candidates. PAS and kinase domains were established for each PERK proteins using CD-Search (Marchler-Bauer and Bryant, 2004).

### Protein Sequence Alignment and Visualization of Putative PERK Homologs

Alignment of protein sequences was performed using Clustal Omega (Sievers and Higgins, 2018) with default parameters in the MEGA (Kumar et al., 1994) interface. Protein alignments were visualized using Jalview (Waterhouse et al., 2009) with Clustalx coloring and sorted by pairwise identity. The Hanging sequences with no conservation, such as was the case for *T. gondii* TgIF2K-A, were removed to the left and right of the alignment in Jalview. Accession numbers for PERK proteins used in alignments were as follows, source organism given in parenthesis: AAS48463 (*Toxoplasma gondii* eIF2α kinase A, also called TgIF2K-A), XP_011239504 (*Mus musculus*), XP_024328881.1 (*Plasmodium falciparum*), XP_953607.1 (*Theileria annulata*), NP_001262283 (*Drosophila melanogaster*), and NP_001300844 (*Homo sapiens*). MycoCosm (Grigoriev et al., 2014) protein Ids for PERK candidates from Neocallimastigomycetes are presented in Supplementary Table 1.

### Construction of Gene Phylogenies for Transmembrane Kinases From Neocallimastigomycetes and Apicomplexa

The following sequences were searched via MMseq2 (Steinegger and Söding, 2017) against the NCBI non-redundant protein database, MycoCosm (Grigoriev et al., 2014), and MMETPS (Keeling et al., 2014): MycoCosm protein Ids 13827 (*Anaeromyces robustus*), 543476 (*Caecomyces churrovis*), 503500 (*Neocallimastix californiae*), and 367815 (*Piromyces finnis*), and NCBI accession numbers XP_024328881.1 (*Plasmodium falciparum*), XP_953607.1 (*Theileria annulata*), AAS48463.1 (*Toxoplasma gondii*), and ACA62938.1 (*Toxoplasma gondii*). For searches of Neocallimastigomycete sequences, the class Neocallimastigomycetes was excluded from the results. Phylogenetic trees were constructed by FastTree (Price et al., 2009) and RAxML (Stamatakis, 2014) from these sequences and their top 100 highest-scoring hits.

### Survey of Heat Shock Proteins From the Fungal Tree of Life

All published genomes in the MycoCosm portal (Grigoriev et al., 2014) were searched for gene models with the annotation KOG0710 Molecular chaperone (small heat-shock protein Hsp26/Hsp42) and KOG0101 Molecular chaperones HSP70/HSC70, HSP70 superfamily. The ratio of the count of gene models annotated as KOG0710 to gene models annotated as KOG0101 was calculated for each genome and averaged over all genomes within each clade (class or phylum). A two-tailed, two-sample unequal variance student's *t*-test (alpha level <0.1) was used to assess significant differences in average hsp ratios between each clade and the Neocallimastigomycetes. The percentage of hsps for each genome was calculated by dividing the sum of all gene models belonging to KOG0101 or KOG0710 by the total number of gene models.

### Routine Cultivation of *Neocallimastix caliorniae* and *Anaeromyces robustus*

The anaerobic fungal strains *Neocallimastix californiae* and *Anaeromyces robustus* were isolated via reed canary grass enrichment from the fecal matter collected from two ruminants at the Santa Barbara Zoo. *N. californiae* originates from a goat; *A. robustus* originates from a sheep. The isolates were separately grown at 39°C under anaerobic conditions in Hungate tubes containing 9.0 mL of autoclaved complex media (“MC”) with 0.1 g of milled reed canary grass as the substrate and 100% CO_2_ in the headspace. The complex media contains 2.5 g/L yeast extract, 6.0 g/L sodium bicarbonate, 10 g/L Bacto™ Casitone, and 15.0 vol% clarified rumen fluid. The fungal strains achieved mid-log phase of growth every 3–4 days and were aseptically transferred at this time point into fresh complex media with 0.1 g of milled reed canary grass as the substrate. Pressure accumulation in the headspace due to the production of fermentation gases was used as a proxy to quantify and track fungal growth.

### Heat-Shock Procedure

1.0 mL of either *N. californiae* and *A. robustus* was inoculated by sterile syringe into 0.1 g of reed canary grass substrate and 10 mL of complex media (Davies et al., 1993) (“MC”) in each Hungate tube with 100% CO_2_ headspace and grown anaerobically at 39°C for 48 h. After the growth period, a total of 24 replicates of each species were subjected to a 48°C water bath for 15 min. The fungi were harvested in replicates of four at 15 min intervals starting immediately before heat shock (control group) up to 60 min after the completion of the heat shock. Standard good practices for working with RNA were followed during all steps. The cultures were transferred from the Hungate tubes to 15 mL Falcon tubes at the time of harvest. They were then centrifuged for 7 min at 4°C and 3,220 g in a swinging bucket rotor (Eppendorf™ A-4-81). One milliliter of RNAlater® (Sigma-Aldrich®) was added to each of the pellets using sterile, filter pipette tips. The samples were then vortexed for 5 s to thoroughly mix the pellet and stabilization solution. The Falcon tubes containing the pellets with RNAlater®, were stored at −20°C until extraction.

### RNA Extraction

For each fungal strain, total RNA from a randomly selected sample from each of four time points was extracted manually to first ensure the presence of high quality RNA. The remainder of the samples were subsequently submitted to automated extraction via a QIAcube (QIAGEN). The frozen cell pellets were thawed from storage on ice and then centrifuged for 10 min at 4°C and 3,220 *g*. The RNAlater™ was decanted from each replicate and the remaining pellets were transferred to previously autoclaved 2-mL screw-cap tubes (Fisher Scientific) containing 1 mL of 0.5 mm zirconia beads (Biospec). To each tube, 450 μL (manual extraction) or 600 μL (QIAcube) of a mixture of buffer RLT (QIAGEN) and 14.3 M β-mercaptoethanol (Sigma) in a ratio of 1 mL to 10 μL, respectively, was added. The cells were lysed using the Biospec Mini-Beadbeater-16 for 1 min, placed briefly on ice, and then centrifuged using a microcentrifuge (Eppendorf™ 5424) for 3 min at room temperature and 13,000 *g*. The lysate was removed using gel-loading pipette tips (Fisher Scientific) and deposited in either round-bottom tubes for total RNA extraction (QIAcube exraction) or QIAshredder tubes (manual extraction). QIAcube extraction was executed following the RNeasy Mini protocol. Manual extraction was completed according to the protocol for “Purification of Total RNA from Plant Cells and Tissues and Filamentous Fungi” outlined in the RNeasy® Mini Handbook. The optional on-column DNAse digest was included in both methods.

### RNA Quality Assessment, Library Preparation, Sequencing, and Data Analysis Pipeline

RNA quality was assessed for the two critical metrics that dictate successful sequencing; concentration by Qubit 2.0 Fluorometer (Invitrogen) and degradation by Agilent TapeStation. All samples exhibited a starting amount of total RNA above the minimum threshold for the sequencing protocol (200 ng) and an RNA Integrity Number (RINe) above 7.0. The Illumina® Truseq® Stranded mRNA kit was used to prepare the mRNA libraries for the *N. californiae* and *A. robustus* samples as it isolates eukaryotic polyadenylated mRNA. The resulting libraries were sequenced into 75 bp single-end reads employing a high output kit to generate more than 400 million reads on an Illumina® NextSeq500. HISAT (Kim et al., 2015) was used to align the reads of each species to their respective genomes, which are publically available for download from the Joint Genome Institute (JGI) MycoCosm portal (Grigoriev et al., 2014). After mapping, featureCounts (Liao et al., 2014) was used to quantify the number of reads mapped to distinct genes for each of the two fungal strains. Subsequently, the DESeq2 (Love et al., 2014) package in R version 3.4.3(R Core Team, 2013) was used to test for differential gene expression between the control and each of the time points following heat shock for *N. californiae* and *A. robustus*, respectively. Genes were classified as differentially regulated if the requirements of an absolute log_2_ fold change ≥1 and a *p*-adjusted value <0.05 were met. The resulting dataset was then analyzed using the functional annotations from KEGG (Ogata et al., 1999), GO, InterPro (Mitchell et al., 2018) and KOG (Koonin et al., 2004) publically available via MycoCosm portal (Grigoriev et al., 2014).

## Data Availability Statement

Protein phylogenies for transmembrane kinases from Apicomplexa and Neocallimastigomycetes are available at the following GitHub repository: https://github.com/cswift3/stress_response_anaerobic_fungi. FASTQ files for all samples sequenced as part of this work available through the National Center for Biotechnology Information (NCBI) BioProject PRJNA665745 at https://dataview.ncbi.nlm.nih.gov/object/PRJNA665745?archive=sra.

## Author Contributions

CS, NM, and MO'M designed the study and analyzed the data and edited the manuscript. CS and NM carried out fungal stress response experiments and RNA-extractions. SM, AS, and IG aided in analysis of transcriptomic sequencing data. CS wrote the paper. All authors contributed to the article and approved the submitted version.

## Conflict of Interest

The authors declare that the research was conducted in the absence of any commercial or financial relationships that could be construed as a potential conflict of interest.

## Publisher's Note

All claims expressed in this article are solely those of the authors and do not necessarily represent those of their affiliated organizations, or those of the publisher, the editors and the reviewers. Any product that may be evaluated in this article, or claim that may be made by its manufacturer, is not guaranteed or endorsed by the publisher.
