# Supplementary material for: A Genomic Catalog of Stress Response Genes in Anaerobic Fungi for Applications in Bioproduction
Source: Front Fungal Biol. 2021 Aug 9;2:708358. doi: 10.3389/ffunb.2021.708358 (PMC10512342; doi:10.3389/ffunb.2021.708358)
Supplement: Supplementary file 1 [file Data_Sheet_1.zip › Swift_Supplementary_tablesandfigures.pdf]

Supplementary Material

1 Supplementary Data

Supplementary Dataset S1: Differentially regulated genes of *A. robustus* in response to heat shock (*p*-adjusted <0.05). Log_2 fold change compares expression at each time point compared to a control without heat shock.

Supplementary Dataset S2: Differentially regulated genes of *N. californiae* in response to heat shock (*p*-adjusted <0.05). Log_2 fold change compares expression at each time point compared to a control without heat shock.

2 Supplementary Figures and Tables

2.1 Supplementary Figures

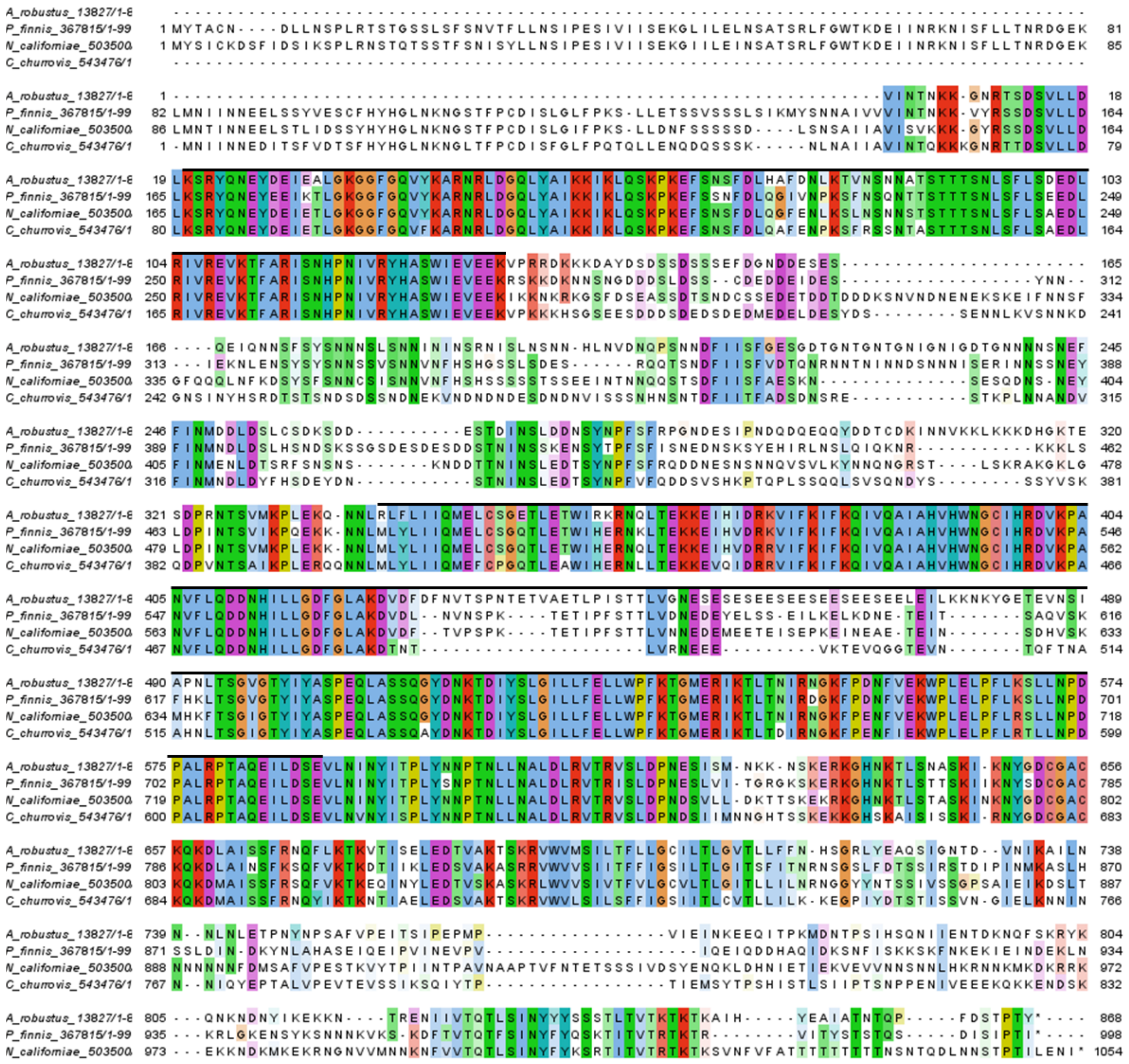

Figure S1: Conserved residues between PERK-like proteins from Neocallimastigomycetes. Black bars indicate protein kinase catalytic domains (Conserved Domain Database(Marchler-Bauer et al., 2014) accession cl21453). Coloring follows Clustal X designations for amino acid properties: Blue=hydrophobic, red=positive, magenta=negative, green=polar, pink=cysteine, orange=glycine, yellow=proline, cyan=aromatic. A threshold of 30% conservation was used to set transparency.

(A)

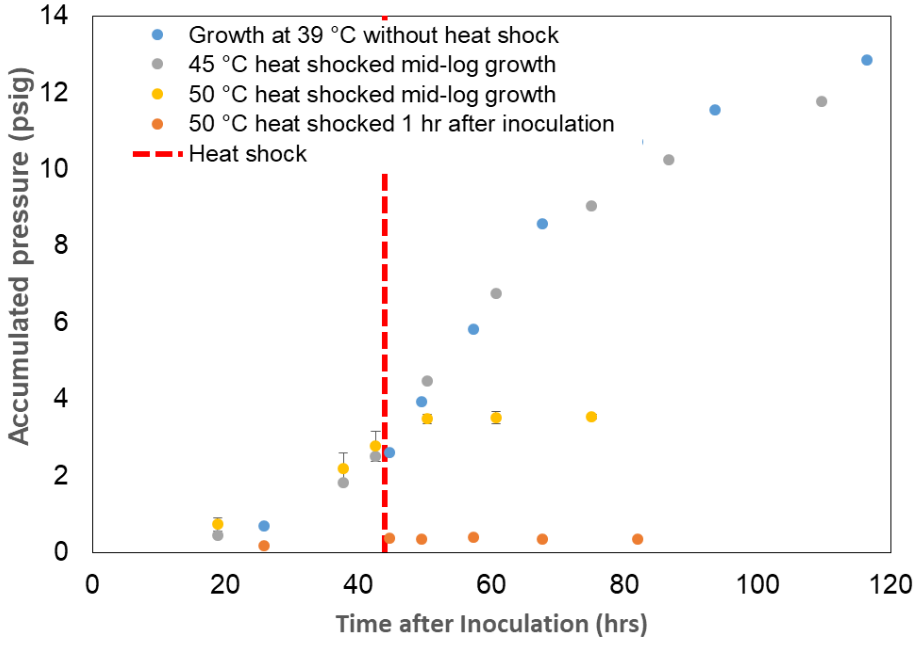
Growth at 39 °C without heat shock
45 °C heat shocked mid-log growth
50 °C heat shocked mid-log growth
50 °C heat shocked 1 hr after inoculation
Heat shock
Accumulated pressure (psig)
Time after Inoculation (hrs)
0
2
4
6
8
10
12
14
0
20
40
60
80
100
120

(B)

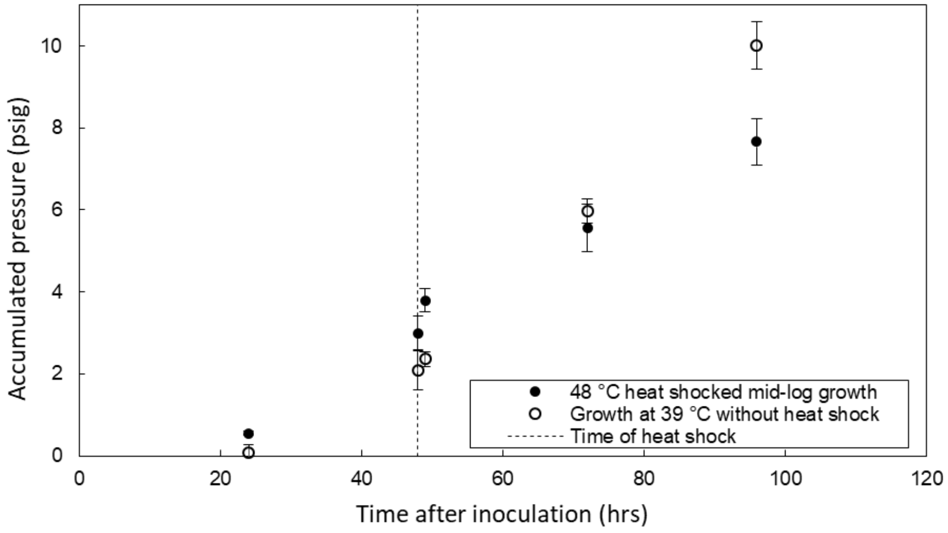
48 °C heat shocked mid-log growth
Growth at 39 °C without heat shock
Time of heat shock
Accumulated pressure (psig)
Time after inoculation (hrs)
0
2
4
6
8
10
0
20
40
60
80
100
120

(C)

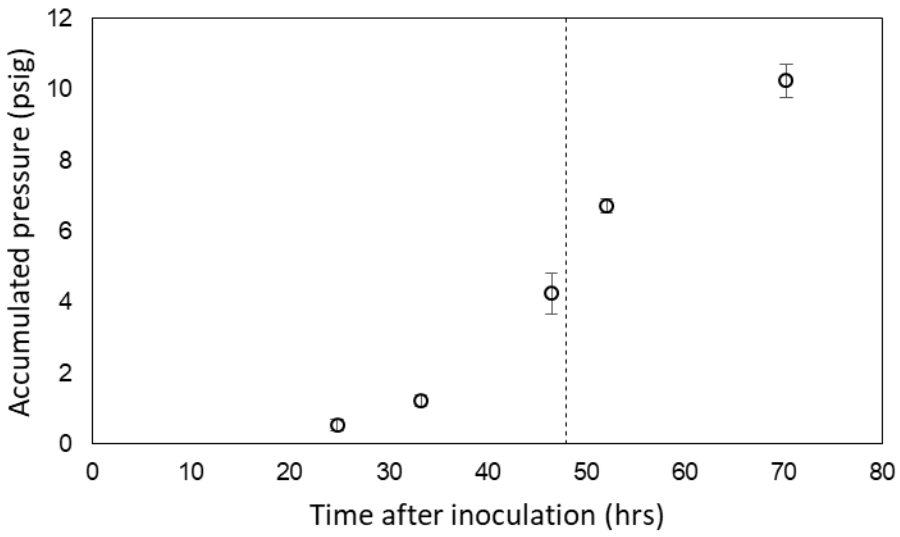

Supplementary Figure S2: Growth of *Neocallimastix californiae* and *Anaeromyces robustus* when exposed to transient heat shock. Growth was assessed by the pressure transducer method (Theodorou et al., 1995). Vertical dashed lines indicate the time of heat shock, excluding the condition of heat shock of 50 °C at 1 hr after inoculation. Each data point represents the average of three biological replicates. (A) *N. californiae* heat shock at varying temperature (1-hr duration), (B) *A. robustus* heat shocked at 48 °C (15-min duration), (C) *N. californiae* cultures harvested for RNA-sequencing with heat shock of 48 °C (15-minute duration).

(A)

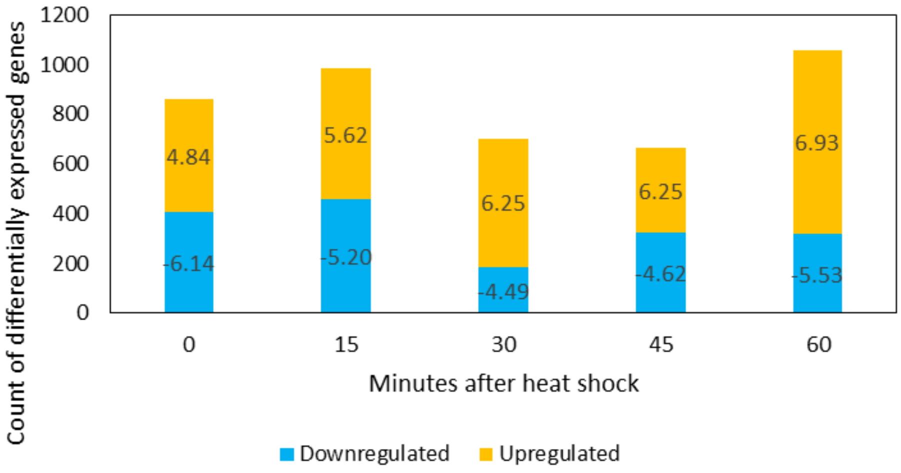

(B)

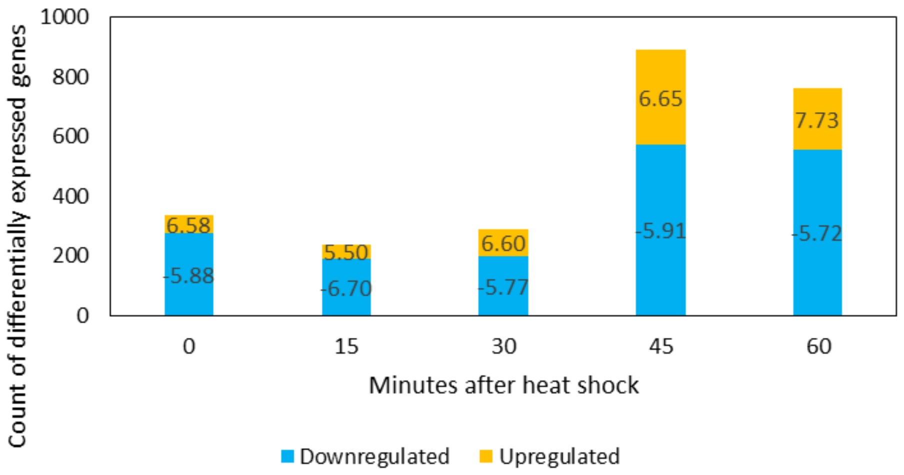

Supplementary Figure S3: Dynamics of the transcriptional response to heat shock. (A) *N. californiae*, (B) *A. robustus*. Upregulated genes have a $\log_2$fold change greater than one relative to before heat shock and downregulated genes have a $\log_2$fold change less than one relative to before heat shock (*p*-adjusted ≤ 0.05). Data labels are the maximum ($\log_2$fold change ≥ 1) or minimum ($\log_2$fold change ≤ 1) values of the most regulated gene at each time point.

2.2 Supplementary Tables

Supplementary Table S1: Key machinery of the unfolded protein response in Neocallimastigomycetes and homology to model organisms. Protein Ids are listed from MycoCosm (Grigoriev et al., 2014). E-value for all results was less than 10^{-8}. Accession numbers used as query sequences are as follows: KAR2 (NP_012500), IRE1 (NP_011946.1), PERK (NP_649538), eIF2alpha (NP_001285329), and ERO1 (NP_013576), CNE1 (NP_009343), and CALR (NP_001262430).

Fungus	Protein Id	% Identity	% Subject Coverage	Regulated[1]	RPKM[2]
**KAR2, *S. cerevisiae***					
A. robustus	292878	69.2	89.0	No	29.06
C. churrovis	594704	52.4	45.4	N/A	N/A
N. californiae	377732	69.3	89.1	Upregulated	22.79
N. californiae	378759	69.3	89.1	No	37.56
P. finnis	579097	69.5	89.1	N/A	N/A
**IRE1, *S. cerevisiae***					
A. robustus	260887	56.3	80.4	No	1.81
C. churrovis	496540	50.7	44.7	N/A	N/A
N. californiae	384816	50	42.9	No	1.19
N. californiae	412463	58	82.1	No	4.45
P. finnis	301873	54.0	78.2	N/A	N/A
**PERK, *D. melanogaster***					
A. robustus	13827/182287[3]	49.1	25.6	No	16.03[3]
C. churrovis	543476	48.2	24.4	N/A	N/A
N. californiae	503500	48	21.2	No	5.04

P. finnis	367815	49.8	22.2	N/A	N/A
**eIF2alpha, *D. melanogaster***					
A. robustus	261532	49.6	83.4	No	8.02
C. churrovis	517625	49.2	83.4	N/A	N/A
N. californiae	500664	48.4	79.5	No	21.28
N. californiae	523066	50.4	84.9	Downregulated	2.55
P. finnis	581387	49.3	78.6	N/A	N/A
**ERO1, *S. cerevisiae***					
A. robustus	230787	43.7	43.5	No	2.69
A. robustus	231651	46.5	41.2	No	N/A
C. churrovis	486941	41.1	47.8	N/A	N/A
C. churrovis	522184	44.6	41.0	N/A	N/A
N. californiae	517753	45.8	42.5	No	Not transcribed
N. californiae	450298	44.0	41.9	No	3.20
N. californiae	428806	43.9	51.8	No	2.17
P. finnis	402783	45.8	41.7	N/A	N/A
P. finnis	323943	46.2	43.4	N/A	N/A
**CNE1 (calnexin), *S. cerevisiae***					
A. robustus	221501	40.8	70.8	No	58.87
C. churrovis	422995	43.0	64.3	N/A	N/A
N. californiae	522025	41.0	70.9	No	17.55
N. californiae	508661	41.0	70.0	No	3.29
P. finnis	580920	43.8	52.5	N/A	N/A
**CALR (calreticulin), *D. melanogaster***					
A. robustus	232658	52.9	70.4	No	30.84
C. churrovis	432815	53.6	65.6	N/A	N/A
N. californiae	674626	53.8	69.7	No	34.55
P. finnis	580787	53.3	74.3	N/A	N/A

[1]Genes that were differentially regulated in heat-shocked cultures of *A. robustus* or *N. californiae* relative to respective cultures without heat shock.

[2]Gene expression (RPKM, reads per kilobase per million mapped reads) of heat-shocked cultures of *A. robustus* and *N. californiae* 60 minutes after completion of heat shock averaged over four biological replicates. Genes with less than 10 raw counts on average were considered not transcribed.

[3]Protein Id 13827 and 182287 are overlapping gene models. Gene expression (RPKM) is reported for protein Id 182287.

Supplementary Table S2: Orthologous genes of *A. robustus* and *N. californiae* upregulated by heat shock. Orthologs were identified by a bidirectional BLAST (Stephen F. Altschul, Warren Gish, Webb Miller, Eugene W. Myers, 1990).

***A. robustus* protein Id**	***N. californiae* protein Id**	**Annotation**
291613	450301	E.C. 2.1.1.45: thymidylate synthase
203618	464587	KOG0046: Ca2+-binding actin-bundling protein (fimbrin/plastin), EF-Hand protein superfamily
262179	699677	MEROPS S09.UPW unassigned peptidase
268473	240549	KOG1124: FOG: TPR repeat

Supplementary Table S3: ***N. californiae*** **genes upregulated by heat shock indicative of UPR/HSR.** Log_2 fold change of the gene expression at 60 minutes after completion of heat shock relative to control with heat shock. Adjusted *p*-value less than or equal to 0.05. Annotations refer to the eukaryotic Orthologous Groups (KOGs) (Koonin et al., 2004), InterPro (Mitchell et al., 2018), or Pfam (Finn et al., 2010) and are available from the MycoCosm portal (Grigoriev et al., 2014).

ProteinId	Log2FC	KOG Annotation
	**Intracellular trafficking, secretion and vesicular transport**	
448600	2.41	Lectin VIP36, involved in the transport of glycoproteins carrying high mannose-type glycans
666096	2.10	Annexin
702979	2.37	ER-Golgi vesicle-tethering protein p115
520343	4.57	Synaptic vesicle protein EHS-1 and related EH domain proteins
678035	2.33	GTP-binding protein SEC4, small G protein superfamily, and related Ras family GTP-binding proteins
450301	2.17	Endocytosis/signaling protein EHD1
462258	2.03	Endocytosis/signaling protein EHD1
459874	2.00	GTPase Rab11/YPT3, small G protein superfamily
321299	1.77	GTP-binding ADP-ribosylation factor Arf6 (dArf3)
698286	1.51	Protein involved in membrane traffic (YOP1/TB2/DP1/HVA22 family)
636991	1.48	ER-Golgi vesicle-tethering protein p115
696904	2.23	ER-Golgi vesicle-tethering protein p115
673754	2.09	Vesicle coat complex AP-3, beta subunit
416162	2.04	ER-Golgi vesicle-tethering protein p115
672010	1.80	ER-Golgi vesicle-tethering protein p115
667258	1.74	Annexin
	**Protein turnover**	
678702	3.54	Subtilisin-related protease/Vacuolar protease B
702757	2.33	Ubiquitin-conjugating enzyme (PF00179)
702758	1.93	Ubiquitin-like proteins
700100	1.92	Ubiquitin-like proteins
454694	1.81	Ubiquitin and ubiquitin-like proteins
701610	1.73	Subtilisin-related protease/Vacuolar protease B
381558	1.48	Ubiquitin and ubiquitin-like proteins
461227	1.10	Ubiquitin-conjugating enzyme (PF00179)
507800	1.01	Subtilisin-related protease/Vacuolar protease B

Supplementary Table S4: *A. robustus* genes upregulated by heat shock indicative of UPR/HSR. Log_2 fold change of the gene expression at 45 minutes after completion of heat shock relative to control with heat shock. Adjusted *p*-value less than or equal to 0.05. Annotations refer to the eukaryotic Orthologous Groups (KOGs) (Koonin et al., 2004), InterPro (Mitchell et al., 2018), or Pfam (Finn et al., 2010) and are available from the MycoCosm portal (Grigoriev et al., 2014). Genes specifically discussed in the main text are emphasized in italics.

ProteinId	Log2FC	KOG Annotation
**Intracellular trafficking, secretion, and vesicular transport**		
291613	5.52	Endocytosis/signaling protein EHD1
297960	2.06	Transport protein particle (TRAPP) complex subunit
294812	1.70	Nuclear transport receptor Karyopherin-beta2/Transportin (importin beta superfamily)
299217	1.68	Karyopherin (importin) alpha
249042	1.53	GTPase Rab6/YPT6/Ryh1, small G protein superfamily
328807	1.48	Sorting nexin SNX11
239866	1.42	Clathrin adaptor complex, small subunit
325124	1.37	GTP-binding ADP-ribosylation factor-like protein yARL3
261239	1.24	Golgi protein
325616	1.22	Prolactin regulatory element-binding protein/Protein transport protein SEC12p
289472	1.22	Nuclear transport receptor Karyopherin-beta2/Transportin (importin beta superfamily)
287286	1.18	Karyopherin (importin) beta 1
293071	1.17	*Vesicle coat complex COPI, beta' subunit*
271324	1.17	Vacuolar sorting protein VPS24
280100	1.14	GTPase Rab1/YPT1, small G protein superfamily, and related GTP-binding proteins
2608481	1.14	Septin family protein (P-loop GTPase)
271187	1.13	Septin family protein (P-loop GTPase)
292264	1.11	Annexin
211639	1.10	Synaptobrevin/VAMP-like protein SEC22
241413	1.04	SNARE protein YKT6, synaptobrevin/VAMP syperfamily
294106	1.04	**Vesicle coat complex COPI, gamma subunit**
276638	1.03	Vesicle coat complex AP-1/AP-2/AP-4, beta subunit
**Protein turnover**		
288193	1.76	26S proteasome regulatory complex, ATPase RPT3
225527	1.55	26S proteasome regulatory complex, ATPase RPT3
222683	1.30	Ubiquitin-specific protease UBP14
324815	1.42	20S proteasome, regulatory subunit beta type PSMB1/PRE7
292040	1.40	Ubiquitin and ubiquitin-like proteins
292798	1.33	Ubiquitin-like protein
197276	1.30	Ubiquitin activating enzyme UBA1
222683	1.30	Ubiquitin-specific protease UBP14
236994	1.24	Ubiquitin-conjugating enzyme (PF00179)
275087	1.21	20S proteasome, regulatory subunit beta type PSMB4/PRE4
285507	1.21	26S proteasome regulatory complex, subunit RPN9/PSMD13

264651	1.18	20S proteasome, regulatory subunit beta type PSMB7/PSMB10/PUP1
**ProteinId**	**Log2FC**	**KOG Annotation**
200113	1.16	26S proteasome regulatory complex, ATPase RPT5
112561	1.12	26S proteasome regulatory complex, subunit RPN7/PSMD6
269892	1.11	Ubiquitin-conjugating enzyme (PF00179)
228527	1.11	Ubiquitin-conjugating enzyme E2
267208	1.09	Tripeptidyl peptidase II
329084	1.08	20S proteasome, regulatory subunit alpha type PSMA2/PRE8
242802	1.02	20S proteasome, regulatory subunit alpha type PSMA7/PRE6
292679	1.01	Ubiquitin-conjugating enzyme (IPR015368)
262301	1.00	20S proteasome, regulatory subunit beta type PSMB5/PSMB8/PRE2

Supplementary Table S5: Constitutive transcription of small hsps during standard laboratory cultivation. Transcriptomes were previously acquired (Solomon et al., 2016) from a variety of cultivation conditions including growth on grass and soluble sugars**.** The transcriptomes were filtered for hsps by InterProScan annotation (Quevillon et al., 2005) The number of genes encoding hsps in each family were estimated from the gene models in the MycoCosm portal (Grigoriev et al., 2014).

	Anaeromyces robustus	***Neocallimastix californiae***	***Piromyces finnis***
**HSP20**			
Gene model count	52	140	21
Median RPKM	10.22	2.68	6.19
Count of transcripts >0.5 RPKM	49	65	22
**HSP70**			
Gene model count	12	68	24
Median RPKM	71.19	4.41	0.79
Count of transcripts >0.5 RPKM	13	48	21
**HSP90**			
Gene model count	12	18	15
Median RPKM	78.88	55.15	33.54
Count of transcripts >0.5 RPKM	5	12	5

3 References

Finn, R. D., Mistry, J., Tate, J., Coggill, P., Heger, A., Pollington, J. E., et al. (2010). The Pfam protein families database. *Nucleic Acids Res.* 38, D211-22. doi:10.1093/nar/gkp985.

Grigoriev, I. V., Nikitin, R., Haridas, S., Kuo, A., Ohm, R., Otillar, R., et al. (2014). MycoCosm portal: Gearing up for 1000 fungal genomes. *Nucleic Acids Res.* 42, 699–704. doi:10.1093/nar/gkt1183.

Koonin, E., Fedorova, N., Jackson, J., Jacobs, A., Krylov, D., Makarova, K., et al. (2004). A comprehensive evolutionary classification of proteins encoded in complete eukaryotic genomes. *Genome Biol.* 5, R7. doi:10.1186/gb-2004-5-2-r7.

Marchler-Bauer, A., Derbyshire, M. K., Gonzales, N. R., Lu, S., Chitsaz, F., Geer, L. Y., et al. (2014). CDD: NCBI's conserved domain database. *Nucleic Acids Res.* 43, D222-226. doi:10.1093/nar/gku1221.

Mitchell, A. L., Attwood, T. K., Babbitt, P. C., Blum, M., Bork, P., Bridge, A., et al. (2018). InterPro in 2019: improving coverage, classification and access to protein sequence annotations. *Nucleic Acids Res.,* 1–10. doi:10.1093/nar/gky1100.

Quevillon, E., Silventoinen, V., Pillai, S., Harte, N., Mulder, N., Apweiler, R., et al. (2005). InterProScan: Protein domains identifier. *Nucleic Acids Res.* 33, 116–120. doi:10.1093/nar/gki442.

Solomon, K. V., Haitjema, C. H., Henske, J. K., Gilmore, S. P., Borges-Rivera, D., Lipzen, A., et al. (2016). Early-branching gut fungi possess a large, comprehensive array of biomass-degrading enzymes. *Science (80-.).* 351, 1192–1195. doi:10.1126/science.aad1431.

Stephen F. Altschul, Warren Gish, Webb Miller, Eugene W. Myers, D. J. L. (1990). Basic Local Alignment Search Tool. *J. Mol. Biol.* 215, 403–410. doi:10.1016/S0022-2836(05)80360-2.

Theodorou, M. K., Davies, D. R., Nielsen, B. B., Lawrence, M. I. G., and Trinci, A. P. J. (1995). Determination of growth of anaerobic fungi on soluble and cellulosic substrates using a pressure transducer. *Microbiology* 141, 671–678. doi:10.1099/13500872-141-3-671.
